# Social Media Mindsets and Well-Being in Emerging Adults: A Serial Mediation of Facebook Addiction and Stress

**DOI:** 10.3390/brainsci15030301

**Published:** 2025-03-12

**Authors:** Dariusz Krok, Magdalena Półtorak

**Affiliations:** 1 Institute of Psychology, University of Opole, 45-040 Opole, Poland; 2 Doctoral School, University of Opole, 45-040 Opole, Poland; 119752@student.uni.opole.pl

**Keywords:** social media mindsets, well-being, Facebook addiction, stress, emerging adults

## Abstract

Background/Objectives: Emerging adults live in a time of rapid technological change, with social media playing a central role in their daily lives. While frequent use of social media is linked to reduced well-being, it also supports personal growth and the pursuit of individual goals. This study aims to identify the relationships between attitudes toward social media and psychological well-being in the context of the mediating roles of Facebook addiction and perceived stress in emerging adulthood. Methods: The study included 294 participants, with a mean age of 23.76 years (SD = 3.23). The following tools were used to measure the variables: the Social Media Mindsets Scale (translated by the authors), the Bergen Facebook Addiction Scale, the Perceived Stress Scale, the Psychological Well-Being Scale, and the Satisfaction with Life Scale. Correlation and mediation analyses were conducted. Results: The findings confirmed most of the assumptions. Attitudes toward social media were positively correlated with psychological well-being. Furthermore, Facebook addiction and perceived stress serve as serial mediators between attitudes toward social media and well-being. Conclusions: Based on the results, practical interventions can be developed to prevent addictions and reinforce stress management, which will help young people maintain their well-being and mental health.

## 1. Introduction

Emerging adulthood, a term coined by Arnett [1], is a distinct developmental period within society. It typically spans from late adolescence to the mid-20s, when individuals are emotionally unstable, seeking their identity, and viewing the world as full of opportunities for personal development [2]. Furthermore, emerging adulthood corresponds to Generation Z. Many studies focus on Generation Z due to their time spent online [3], a sense of uncertainty [4], and other associated characteristics and values. As previous generations did not have such accessible technology to use at any moment, they learned to live in a new reality. In contrast, Generation Z naturally incorporates media into their daily lives, shops online, forms relationships on social media, shares their experiences and thoughts in the virtual space, and even treats it as a form of escape from reality. This may serve as a form of coping strategy directed at creating distance from life concerns [5]. Young peoples’ daily functioning and mindset are influenced by the rapid development of technology and social media, which have become some of the most significant aspects of life today. Easy access to the Internet results in them spending more and more time in the virtual world. Characterising this generation, Twenge [4] noted that fewer in-person meetings and reduced interactions and conversations, even with close family members, contribute to increased feelings of loneliness, symptoms of depression, and anxiety. Consequently, the younger generation is increasingly unhappy [4].

This article focuses on young people’s subjective approach to social media, examining the relationship of beliefs about social media with psychological well-being in the context of Facebook addiction and perceived stress. It consists of five main sections: Section 1, which provides information about the studied variables and key theories; Section 2, describing the participants, research tools, study procedure, and data analysis approach; Section 3, presenting the findings from statistical analyses; Section 4, interpreting our results in light of hypotheses and theories; and Section 5, summarising the key takeaways from the study. Our study expands on the existing literature by highlighting that Facebook addiction and stress can act as significant mediators between attitudes toward social media, psychological well-being, and life satisfaction. Additionally, it emphasises the significance of psychological mechanisms related to an individual’s mental attitudes (such as addiction and stress) in shaping psychological well-being.

### 1.1. Social Media Mindsets and Psychological Well-Being

According to Gollwitzer and Keller [6], mindset theory states that before taking action, individuals consider and decide how important the goal they wish to achieve is, why they want to achieve it, and whether they have the appropriate predispositions to accomplish it. The Model of Action Phases (MAP) framework, a theoretical model developed by Gollwitzer [7], identifies four stages of action influenced by two types of mindsets: deliberative and implemental. The deliberative mindset is based on a cognitive, thorough evaluation of the advantages and disadvantages of goals and actions. It is characterised by openness to new information to ensure the best possible outcome of the action [8]. In contrast, the implemental mindset emphasises determination and focused attention on strategies to help achieve the intended goal [9].

Social media mindsets refer to the general stance and beliefs about media and their role in the life of a modern individual [10]. It stems from the perception of how much control a person feels over media and whether they perceive the influence of media on their life as positive or negative [11]. The study by Lee and Hancock [10] indicates that this attitude consists of agency and valence. The agency dimension refers to the sense of control over the use of social media, including the frequency and intensity of contact with it. In contrast, the valence dimension describes the positive or negative attitude toward the media [10]. An individual who views social media positively is convinced that it helps maintain personal relationships, provides more significant opportunities for personal development, and facilitates information acquisition. A person with a negative attitude toward social media believes using it is a waste of time and carries many risks. This understanding of individual control can empower individuals to make conscious choices about their social media use, influencing their well-being.

How a person perceives social media affects the actions related to it. In turn, actions that are meaningful to individuals and allow them to grow can enhance their satisfaction with life and well-being. Although some researchers treat these variables as synonyms, using them interchangeably (e.g., Prapas and Mayreas [12]), it is still worth pointing out the main difference between them. Satisfaction with life is a subjective feeling of contentment with one’s current life situation and a positive evaluation of various aspects of life. In Diener’s concept [13], satisfaction with life results from an overall assessment of reality and is rather a superficial element. A more comprehensive evaluation of one’s life is well-being—a multidimensional construct related to a deeper sense of life purpose, personal values, and happiness [14].

Ryff’s [15] concept of psychological well-being emphasises the importance of the feelings accompanying personal growth and the realisation of one’s potential. Well-being is not merely associated with momentary positive affect and hedonic satisfaction but highlights aspects of life that contribute to the holistic growth of an individual. When used positively and controlled, social media can contribute to self-acceptance and personal growth. Ryff [15] operationalises the dimensions of psychological well-being: self-acceptance (the ability to accept oneself, including one’s positive and negative qualities), positive relations with others (the ability to form and maintain satisfying and supportive relationships), autonomy (the ability to make independent decisions and have control over one’s life), environmental mastery (the ability to manage and adapt to the environment), purpose in life (having goals and a sense of direction), and personal growth (the sense of continued development and improvement). According to the author, these dimensions are essential in human life, contributing to life satisfaction and self-contentment and reducing the risk of mental disorders.

Although social media facilitates life and communication with others, many studies indicate that it affects well-being and mental health [16]. Social media can serve as a coping mechanism for everyday life. As Bellini et al. [17] suggested, social media is used to escape from reality. Using a smartphone as a communication tool carries the phenomenon of attention distraction, and being absorbed by virtual reality can be considered a pathological strategy for emotion regulation [17]. Andreassen et al. [18] showed that the addictive use of social networking sites was positively associated with emotional disorders such as anxiety and depression. Time spent on Facebook was positively related to the development of depressive and anxiety symptoms in adolescents [19]. A meta-analysis conducted by Hancock et al. [20] also indicated that anxiety and depression are manifestations of excessive social media use. A study by Lee and Hancock [10] revealed that individuals who believe in having control over their use of social media benefit from it and do not experience psychological stress, in contrast to those whose social media use gets out of control. The research by Wilcox and Stephen [21] showed that using social media can lead to a decrease in self-control over one’s behaviour. As Klein [22] suggests, self-control plays an important role in the context of social media usage. There are negative relationships between social media use and self-control, as well as between self-control and well-being.

In addition, both dimensions of social media mindsets, valence and agency, were negatively related to psychological distress, anxiety, depression, and stress in a multinational sample. Valence, but not agency, was also positively related to life satisfaction [11].

### 1.2. The Potential Mediating Role of Facebook Addiction and Perceived Stress

Social media can serve specific social, informational, cultural, or entertainment purposes. Interacting with others through social media or acquiring desired information can contribute to individual satisfaction and fulfilling needs. The situation becomes more complex when using social media turns into a dominant activity. It is crucial to understand the potential risks and benefits of social media use and manage it to promote well-being. The easy and rapid access to information has led to increasingly frequent use of messaging platforms and social media sites. The necessity of using social media has become a contributing factor to the development of addiction. However, by being aware of these risks and actively managing their social media use, individuals can maintain a healthy balance and promote their well-being. Statistics indicate that an increasing number of people are at risk of becoming addicted to social media use, particularly during emerging adulthood, as nearly everyone in this age group interacts with the Internet [23].

Research has shown the presence of mediating factors between attitudes toward social media and psychological well-being. Liu and Yu [24] found that using Facebook was not directly related to well-being but was mediated by other factors, such as social support. Lee and Hancock [10] demonstrated that active and passive social media use mediate between attitudes toward social media and psychological well-being. Furthermore, research showed a significant connection between Internet use, including social media, and the experience of stress [25]. It is partly due to the mechanism of social comparisons [26]. People using media see the profiles of others and compare their lives to the ones they present, which can cause stress, especially when there is a negative comparison. Moreover, as reports indicate, experiencing stress lowers the level of happiness, while interventions aimed at stress reduction increase satisfaction [27]. The large amount of information individuals receive through intensive social media use increases stress [28]. As a result of an excessive amount of information, information overload occurs, which is a constant cause of experiencing intense stress and exhaustion [29]. Experiencing stress, in turn, has a significant impact on mental health, leading to the development of disorders such as anxiety and depressive disorders [24], thereby reducing life satisfaction and psychological well-being [30,31].

Facebook addiction is a form of behavioural addiction that is associated with excessive, uncontrolled use of the platform, often at the expense of other life activities [32]. This addiction can result from positive reinforcement, such as quick rewards in the form of likes, comments, and online interactions, which activate reward mechanisms in the brain [33]. Pathological Internet use, as described by Davis [34], was associated with lowered self-esteem, weakened relationships with others, and an exacerbation of psychopathological symptoms. Internet addiction was correlated with reduced psychological well-being [35]. Moreover, there were mediating factors in the relationship between these variables. Iyer et al. [35] demonstrated the mediating role of online/offline integration and self-compassion between Internet addiction and aggression, as well as between Internet addiction and psychological well-being.

Excessive use of Facebook as an online social platform may have similar consequences. This view is supported by research showing that social media addiction is negatively correlated with mental well-being and life satisfaction [36,37], thus contributing to the development of various mental health issues, such as depression and anxiety symptoms [38].

Research suggests that Facebook addiction is moderately correlated with daily experiences of stress [39,40,41]. Satici [42] demonstrated that Facebook addiction was negatively correlated with life satisfaction. Individuals addicted to this platform were less happy and more often experienced negative emotions. Additionally, research highlights the presence of mediating factors linking Facebook addiction to well-being. For example, Satici [42] reported that factors such as loneliness and shyness contributed to the more significant negative impact of Facebook addiction on levels of subjective well-being. Akin and Akin [43] studied the relationships between Facebook addiction and life satisfaction, and their findings also revealed the existence of a mediator−social safety. These studies indicate the presence of mediating factors between Facebook addiction and well-being.

**Figure 1:**
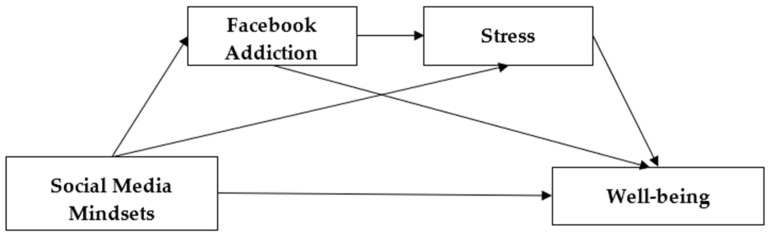
A model of the serial mediation of Facebook addiction and stress and their relationships with social media mindsets and well-being.

The main aim of the present study was to examine the relationship of social media mindsets with well-being (psychological well-being and life satisfaction) among emerging adults within a serial mediation model of Facebook addiction and stress (Figure 1). Based on mindset theory and previous empirical findings, we hypothesised that social media mindsets would be positively related to psychological well-being and life satisfaction and negatively related to Facebook addiction and stress. We also hypothesised that Facebook addiction would be positively related to stress. Finally, we hypothesised that Facebook addiction and stress would serially mediate the relationship of social media mindsets with psychological well-being and life satisfaction among emerging adults, respectively.

## 2. Materials and Methods

### 2.1. Participants

The final sample consisted of 294 participants in the developmental period of emerging adulthood (18−29 years old). The sample comprised 138 men (46.9%) and 156 women (53.1%), with a mean age of 23.76 years (SD = 3.23). Regarding the level of education, seven individuals had primary education, 9 had basic vocational education, 158 had secondary education, and 128 had university education. Criteria for inclusion were as follows: (a) age within emerging adulthood, (b) cognitive capacity to fill in questionnaires, and (c) lack of cognitive deficiencies (e.g., serious memory problems, medical history of psychiatric disorders) that could likely distort responses. The data were collected in southern parts of Poland.

### 2.2. Procedure

Volunteers who met the above inclusion criteria were invited to participate in the study, and the research assistant consequently contacted those who agreed. They were provided with general information about the study’s purpose and procedures and then given a set of questionnaires to be completed in their own time. Written informed consent was obtained to guarantee confidentiality and clarify the study’s purpose. Participants did not receive any financial reimbursement for their involvement. In case of any queries, the research assistant was available to provide clarification. The research protocol was approved by The University Research Ethics Committee at the University of Opole (Approval Code: UREC 65/2024) and fully adhered to the guidelines in the Declaration of Helsinki.

### 2.3. Measures

#### 2.3.1. Social Media Mindsets

The Social Media Mindsets Scale [10] was used to assess individuals’ expectations, behaviours, attributions, and goals about social media’s role in their lives. The scale includes twelve items, to which participants respond on a 5-point Likert scale, ranging from 1 (strongly disagree) to 5 (strongly agree). The scores are calculated on two subscales: (1) valence—which represents people’s beliefs that the effects of social media are beneficial or harmful (e.g., “Using social media is meaningful for me” or “Using social media is fun and enjoyable for me”), and (2) agency—which reflects people’s feelings and opinions that social media use is under their control (e.g., “I’m good at managing the ways I use social media” or “I’m in control of how I use social media”). Higher scores point to more positive valence beliefs and higher perceived agency. The reliability coefficients for the present study were Cronbach’s alpha = 0.82 and McDonald’s omega = 0.82 for valence, and Cronbach’s alpha = 0.74 and McDonald’s omega = 0.71 for agency.

#### 2.3.2. Facebook Addiction

The Bergen Facebook Addiction Scale [44] was applied to measure the level of Facebook addiction. It includes six items assessed on a 5-point Likert scale, ranging from 1 (very rarely) to 5 (very often). These items reflect the following six addiction criteria: 1—dominance, 2—tolerance, 3—mood change, 4—relapse, 5—withdrawal symptoms, 6—conflict (e.g., “You spend a lot of time thinking about social media or planning how to use it” or “You feel an urge to use social media more and more”). Higher scores reflect higher levels of Facebook addiction. Previous research demonstrated that the scale is a valid and reliable method of measuring Facebook addiction among young people [30]. The reliability coefficients for the present study were Cronbach’s alpha = 0.83 and McDonald’s omega = 0.82.

#### 2.3.3. Stress

The Perceived Stress Scale (PSS-10) [45] is a widely used tool that measures the level of stress experienced by individuals in various life domains. The scale assesses the degree to which people perceive life as unpredictable, uncontrollable, and overburdening (e.g., “In the last month, how often have you been upset because of something that happened unexpectedly” or “In the last month, how often have you felt nervous and stressed?”). The scale includes ten items, each answered on a 5-point scale ranging from 0 (never) to 4 (very often). Higher scores indicate a higher intensity of perceived stress. The reliability coefficients for the present study were Cronbach’s alpha = 0.84 and McDonald’s omega = 0.83.

#### 2.3.4. Psychological Well-Being

The short version of the Psychological Well-Being Scale (PWB) [46] was used to measure an individual’s well-being level regarding personal growth, development, and self-realisation. It comprises eighteen items assessed on a 6-point Likert scale, ranging from 1 (strongly disagree) to 6 (strongly agree). The scale contains six subscales: (1) autonomy—which reflects people’s self-determination and independence as well as an ability to resist social pressures (e.g., “My decisions are not usually influenced by what everyone else is doing”), (2) environmental mastery—which describes one’s sense of mastery and competence in managing the environment and controlling multifaceted activities (e.g., “In general, I feel I am in charge of the situation in which I live”), (3) personal growth—which represents a feeling of continued development and expanding one’s potential (e.g., “With time, I have gained a lot of insight about life that has made me a stronger, more capable person”), (4) positive relations with others—which reflects satisfying and trusting relationships with others (e.g., “Most people see me as loving and affectionate”), (5) purpose in life—which defines a feeling of having clear goals in life and a sense of directedness (e.g., “I have a sense of direction and purpose in life”), and (6) self-acceptance—which reflects a positive and affirmative attitude toward the self (e.g., “When I look at the story of my life, I am pleased with how things have turned out”). Their sum gives the total score. Higher scores reflect a higher level of psychological well-being. Due to the purpose of the current study and the psychometric recommendations, only the total score was used for statistical calculations [46,47]. The reliability coefficients for the present study were Cronbach’s alpha = 0.88 and McDonald’s omega = 0.87.

#### 2.3.5. Life Satisfaction

The Satisfaction with Life Scale (SWLS) [48] was applied to gauge overall cognitive judgments of satisfaction with one’s life. It assesses cognitive rather than emotional aspects of life satisfaction (e.g., “In most ways my life is close to my ideal” or “The conditions of my life are excellent”). The scale consists of five items rated on a 5-point Likert scale, ranging from 1 (strongly disagree) to 5 (strongly agree). The overall score is calculated by summarising the scores of individual items. Higher scores indicate more life satisfaction. The reliability coefficients for the present study were Cronbach’s alpha = 0.86 and McDonald’s omega = 0.85.

### 2.4. Data Analysis

First, a priori power analysis was conducted using G*Power to estimate the sample size required for the current study [49]. Its results showed that a minimum of 244 participants was sufficient to obtain a small effect size = 0.05 with the following parameters: power (1 − β) = 0.80 and α = 0.05. Finally, a more significant number of individuals (N = 294) was included to ensure the sample’s representativeness within the population of Polish emerging adults and to minimise potential errors in estimating the effect size. The convergent and discriminant validity of the constructs in this study were verified by calculating the following parameters: the composite reliability (CR) values, the average variance extracted (AVE) values, and factors loadings for each construct. The composite reliability (CR) values were as follows: social media mindsets—valence = 0.76, social media mindsets—agency = 0.70, Facebook addiction = 0.78, stress = 0.76, psychological well-being = 0.82, life satisfaction = 0.80. The average variance extracted (AVE) values were as follows: social media mindsets—valence = 0.51, social media mindsets—agency = 0.52, Facebook addiction = 0.52, stress = 0.54, psychological well-being = 0.52, life satisfaction = 0.53. The factor loadings (λ) of all the items were as follows: social media mindsets—valence = from 0.76 to 0.66, social media mindsets—agency = from 0.62 to 0.75, Facebook addiction = from 0.70 to 0.81, stress = from 0.68 to 0.0.82, psychological well-being = from 0.63 to 0.83, and life satisfaction = from 0.61 to 0.86.

We also checked common method bias by using Harman’s single factor test and variance inflation factor (VIF). Harman’s one-factor test was performed to exclude the possibility of common method variance [50]. The results indicated that all items with the first unrotated factor accounted for 25.49% of the variance. Therefore, common method variance is not present in our study. The amount of multicollinearity in regression analysis was also acceptable—the VIF was 1.95.

Next, the following analyses were conducted using IBM SPSS Statistics, Version 28: (1) two-tailed correlations to examine initial associations among the study variables; (2) serial mediation analysis to examine whether social media mindsets (the predictor) directly or/and indirectly—via Facebook addiction and stress (the mediators)—affects well-being (outcome variables). As Hayes [51] recommended, Model 6, with bootstrapping of 5000 samples and 95% confidence intervals, was used to calculate both single and serial mediational effects. Missing data were controlled by case-wise mean substitution to eliminate potential statistical miscalculations.

## 3. Results

### 3.1. Initial Correlations Among Variables

First, Pearson correlation coefficients were used to examine the relationships among the study variables: social media mindsets (valence and agency), psychological well-being, life satisfaction, Facebook addiction, and stress. The results are presented in Table 1.

A coefficient value analysis showed that valence correlated positively only with psychological well-being. In contrast, agency correlated positively with psychological well-being and life satisfaction and negatively with Facebook addiction and stress. Psychological well-being was positively associated with life satisfaction and negatively associated with Facebook addiction and stress. Life satisfaction was negatively associated with stress. Finally, Facebook addiction was positively associated with stress. All relationships had weak or moderate correlations.

### 3.2. Serial Mediation Analysis

To examine whether Facebook addiction and stress would serially mediate the relationship of social media mindsets with psychological well-being and life satisfaction, respectively, a serial mediation model with bootstrapping was applied (model 6; samples = 5000; 95% bias-corrected confidence intervals) [51]. The dimensions of social media mindsets were valence and agency, the predictor variables; psychological well-being and life satisfaction, the outcome variables; and Facebook addiction and stress scores, the serial mediators.

The results of direct effects and total effects for the study variables are presented in Table 2.

For valence as an independent variable and psychological well-being as a dependent variable, valence was not significantly related to either Facebook addiction or stress. In contrast, Facebook addiction was positively related to stress and negatively related to psychological well-being. Stress was negatively related to psychological well-being. Finally, valence was positively related to psychological well-being. The total effect of valence on psychological well-being was statistically significant.

For agency as an independent variable and psychological well-being as a dependent variable, agency was negatively related to both Facebook addiction and stress. Facebook addiction was positively related to stress and negatively related to psychological well-being, whereas stress was negatively related to psychological well-being. Lastly, agency was not related to psychological well-being. The total effect of agency on psychological well-being was statistically significant.

For valence as an independent variable and life satisfaction as a dependent variable, valence was not significantly related to either Facebook addiction or stress. Facebook addiction was positively related to stress, which, in turn, was negatively related to life satisfaction. There was no significant relationship between valence and life satisfaction. The total effect of valence on life satisfaction was non-significant.

Finally, for agency as an independent variable and life satisfaction as a dependent variable, agency was negatively related to both Facebook addiction and stress. Facebook addiction was positively related to stress, which, in turn, was negatively related to life satisfaction. There was no statistically significant relationship between agency and life satisfaction. In contrast, the total effect of agency on life satisfaction was statistically significant.

Next, indirect effects of social media mindsets on psychological well-being and life satisfaction, through the mediating variables of Facebook addiction and stress, were calculated. They are shown in Table 3.

The results demonstrated that Facebook addiction and stress were serial mediators between agency and psychological well-being and life satisfaction, respectively. A close analysis of the direct results showed that agency was negatively associated with Facebook addiction, which, in turn, was positively associated with stress, consequently leading to a negative relationship with psychological well-being and life satisfaction, respectively. In contrast, no serial mediational effects were found between valence, psychological well-being, and life satisfaction. Taking into account the separate mediational effects, both Facebook addiction and stress were single mediators in the association between agency and psychological well-being. In addition, stress also mediated the relationship of agency with life satisfaction.

Finally, the effect-contrast method was used to test contrasts among mediated effects in all the models. For agency as an independent variable and psychological well-being as a dependent variable, there was no difference between Facebook addiction and stress in their mediating powers (indirect effect = −0.02; CIs [−0.09, 0.05]). However, for agency as an independent variable and life satisfaction as a dependent variable, the effect-contrast results demonstrated differences in the mediating powers of Facebook addiction and stress (indirect effect = −0.11; CIs [−0.20, −0.03]), with stress having a more substantial mediation effect than Facebook addiction.

## 4. Discussion

This study examined relationships between social media mindsets and well-being within a serial mediation model of Facebook addiction and stress in emerging adults. Based on mediational analysis, the findings mostly supported all three hypotheses. To the best of our knowledge, this is the first study to investigate serial mediational pathways between core assumptions about the nature of social media and well-being in a sample of emerging adults. Consequently, it extends mindset theory and provides new knowledge for understanding social media effects in the context of well-being.

### 4.1. Explaining Associations Between Social Media Mindsets and Well-Being

In the first hypothesis, we assumed that social media mindsets would be positively associated with psychological well-being and life satisfaction and negatively associated with Facebook addiction and stress. The hypothesis was partially confirmed, as only the dimension of agency of social media mindsets was associated with all of the above variables. Our findings showed that agency mindsets of social media use were positively correlated with psychological well-being and life satisfaction and negatively associated with Facebook addiction and perceived stress. Individuals who believed in having greater control of their use of social media were more satisfied with life and exhibited better well-being. This is in line with the results obtained by Lee and Hancock [10], indicating that individuals confident in their agency in using social media experienced less psychological stress and were more satisfied with their lives. Research on the association between agency, in general, and the psychological well-being of young people revealed that those who believed in being able to control various aspects of their lives exhibited positive affect, were happier, and more satisfied with life [46]. It suggests that having a sense of control over social media usage is associated with better well-being, life satisfaction, and overall psychological well-being.

In contrast, valence mindsets of social media use were positively associated only with psychological well-being, which is consistent with the findings of Lee and Hancock [10]. People who perceive social media use positively report higher levels of well-being, whereas those with a negative attitude toward social media experience lower levels of well-being.

In the present study, no significant associations were observed between the valence of social media and life satisfaction, Facebook addiction, and stress. It indicates that for the participants, the valence of social media itself does not significantly affect life satisfaction, Facebook addiction, or stressful feelings. This is an interesting result, which points to differences in the approach of young people to the perceived valence of social media’s effect on their lives compared to agency. One potential explanation may refer to the developmental characteristics of the study group—namely, emerging adults who have been exposed to social media from an early age tend to view it as a natural part of life [28,52,53]. Thus, they do not pay too much attention to whether the effects of using social media could be enhancing or harmful for them.

This interpretation is buttressed by previous research on relationships between social media use and well-being, in which social media addiction was linked to the time spent on these platforms [54]. The lack of a significant relationship between valence and stress might also stem from a dual role played by social media: it can increase perceived stress but is also used as a coping mechanism [55]. Therefore, for this group, the time spent on social media or how it is used is likely more significant for life satisfaction and stress levels than the valence of the content.

The second hypothesis assumed that Facebook addiction would be positively associated with stress. Our study confirmed this assumption, which is in line with other studies. Through extensive research on Facebook addiction, Atroszko et al. [39] found that it was positively correlated with stress and negatively associated with quality of life. In addition, it was a significant predictor of experiencing stress in daily life. Individuals struggling with excessive Facebook use tend to experience more intense stress and negative emotions. Similarly, Ho [56] demonstrated a positive correlation between Facebook addiction and stress. People with high levels of stress are more predisposed to addictions, including Facebook addiction. According to the addiction mechanism, stress is often one of the causes of engaging in problematic behaviours [57,58]. As a consequence, emerging adults who overuse Facebook will experience higher stress compared to their peers who do not exhibit this type of behaviour.

### 4.2. Serial Mediational Effects of Facebook Addiction and Stress

The main finding of the current study concerns the third hypothesis, which assumed that Facebook addiction and stress would serially mediate the relationship between social media mindsets and well-being. Our mediation analyses convincingly showed that Facebook addiction and stress were serial mediators only in the relationship of agency with psychological well-being and life satisfaction. The associations between the amount of agency emerging adults feel over their use of social media and well-being are thus not directly related but are rather mediated by psychological factors related to addiction and stress. In contrast, no mediational results were found in the case of valence. This interesting result highlights the difference in the psychological roles played by agency and valence in the spheres of psychological well-being and life satisfaction. Different underlying mechanisms likely exist between both dimensions of social media mindsets in terms of consequences for well-being.

Agency in the context of attitudes towards social media can be explained using Deci and Ryan’s self-determination theory [59]. This theory emphasises that autonomy in decision-making is crucial for an individual’s well-being. In the context of social media, agency refers to the sense of control over how much time a person spends on media, what they post, and how they react to others’ posts. Self-control reduces the risk of addiction, thus helping to maintain good well-being [60].

A sense of agency reduces Facebook addiction and stress, improving psychological well-being and overall life satisfaction. The findings suggest that strengthening agency in young adults may limit the development of Facebook addiction, reduce experienced stress levels, and consequently improve their well-being and overall mental state. Similarly, Błachnio and Przepiórka [61] demonstrated that low levels of self-control can contribute to the development of Facebook addiction. Loss of control is also one of the symptoms indicative of addiction [44], and it simultaneously reduces life satisfaction and contributes to psychological distress [62]. Self-control is one of the factors that influence the development of mental disorders or the maintenance of overall mental well-being. Hofmann et al. [63] demonstrated that individuals with a high level of self-control are satisfied with their lives, and experiencing positive emotions further enhances their satisfaction.

Additionally, the indirect effect of Facebook addiction on stress highlights the significance of problematic Facebook and social media use. Sedera and Lokuge [64] found that stress levels increased among individuals who abstained from social media and remained elevated even after the study ended. This may be attributed to the phenomenon known as Fear of Missing Out, which introduces psychological distress into daily life when social media is not used regularly [65]. Limiting Facebook usage may lead to reduced stress levels and an improvement in life satisfaction and psychological well-being.

The analyses did not reveal significant indirect or total effects regarding the perceived valence of social media. Both Facebook addiction and stress are not significant factors in the relationship between perceived valence and psychological well-being or life satisfaction. It can be assumed that the relationship between valence and well-being is either direct or based on other mechanisms not accounted for in this study. The existing trend in virtual space for people to compare themselves with others plays a more significant role in psychological well-being. According to Festinger’s social comparison theory [66], people compare themselves with others either “upward” or “downward.” The first means we evaluate ourselves as better than others; the second, the opposite. In cases where a person sees an idealised world of others on social media, they may compare themselves “downward”, which can result in a decrease in mood and life satisfaction [26]. Another explanation may refer to personality traits as moderating variables in the relationship between valency mindsets and well-being. Kuss and Griffiths [32] demonstrated that, for example, the level of neuroticism leads to different approaches to social media, which in turn may affect well-being. The multidimensional model of social media use suggests the presence of such social mechanisms as social support, social comparisons, or emotional contagion, which may significantly impact the relationship between valence, well-being, and life satisfaction [52]. Furthermore, Bekalu et al. [67] demonstrated that individuals who use social media for emotional reasons are less likely to experience social well-being. This may indicate that emotional regulation could be a more significant variable.

### 4.3. Limitations and Future Implications

The current study is not devoid of limitations. First, it was conducted within a specific age group: emerging adulthood (18–29 years). Comparative studies involving other age groups could prove interesting, as older generations did not grow up in a virtual world and may exhibit different attitudes compared to young adults. Second, another limitation of the study was the use of a scale measuring Facebook addiction instead of assessing social media addiction in general. This was due to the lack of availability of such a scale in the Polish market. Although, in recent years, young people have mostly abandoned Facebook in favour of Instagram and TikTok, we decided to focus on Facebook as in our cultural context (Poland), there are still many emerging adults who are active on Facebook. Moreover, in our study, we examined only individuals who use Facebook, and one of the scales specifically referred to this group (i.e., The Bergen Facebook Addiction Scale). Future research should focus more on investigating psychological mechanisms of using Instagram and TikTok. Third, our cross-sectional study prevents us from drawing causal conclusions. Longitudinal and experimental studies introducing interventions aimed at examining the role of Facebook addiction and stress could thus be of future interest.

## 5. Conclusions

The study demonstrated the importance of Facebook addiction and stress as significant mediating factors that influence associations between social media mindsets, psychological well-being, and life satisfaction, as well as the underlying mechanisms of social media addiction. Emerging adults who feel they have satisfactory control over social media tend to be less addicted to Facebook, and consequently experience less stress, leading to better mental well-being and life satisfaction. The findings may serve as a basis for designing effective psychological and social interventions in the future, focusing on stress management and addiction prevention, which will help young people maintain their mental health. They also emphasise the relevance of taking into account the psychological mechanisms of addiction and stress in building educational programmes aimed at constructive use of social media by young people.

## Figures and Tables

**Table 1:** Descriptive statistics and correlations among social media mindsets (valence and agency), psychological well-being, life satisfaction, Facebook addiction, and stress in emerging adults (N = 294).

Variables	M	SD	1.	2.	3.	4.	5.
1. Valence	3.45	0.74	−				
2. Agency	3.35	0.80	0.07	−			
3. Psychological well-being	4.41	0.68	0.12 *	0.21 ***	−		
4. Life satisfaction	4.39	1.25	0.05	0.18 **	0.60 ***	−	
5. Facebook addiction	1.97	0.85	0.11	−0.39 ***	−0.28 ***	−0.11	−
6. Stress	2.02	0.69	−0.02	−0.29 ***	−0.44 ***	−0.45 ***	0.20 ***

* *p* < 0.05; ** *p* < 0.01; *** *p* < 0.001.

**Table 2:** Direct effects and total effects for Facebook addiction and stress in the relationship of social media mindsets (valence and agency) with psychological well-being and life satisfaction (standardized scores).

Pathways	Estimate	Standard Error	*t*	Model *R*^2^
Direct effects				
Valence as the Independent Variable and Psychological Well-Being as the Dependent Variable				
Valence—Facebook Addiction	0.11	0.07	1.90	0.01
Valence—Stress	−0.04	0.05	−0.69	
Facebook Addiction—Stress	0.21	0.05	3.58 ***	0.04 **
Facebook Addiction—Psychological Well-Being	−0.22	0.04	−4.21 ***	
Stress—Psychological Well-Being	−0.38	0.05	−7.45 ***	
Valence—Psychological Well-Being	0.14	0.04	2.68 **	0.24 ***
Agency as the Independent Variable and Psychological Well-Being as the Dependent Variable				
Agency—Facebook Addiction	−0.39	0.06	−7.32 ***	0.15 ***
Agency—Stress	−0.25	0.05	−4.15 ***	
Facebook Addiction—Stress	0.10	0.05	1.99 *	0.10 ***
Facebook Addiction—Psychological Well-Being	−0.20	0.05	−3.55 ***	
Stress—Psychological Well-Being	−0.39	0.05	−7.21 ***	
Agency—Psychological Well-Being	0.02	0.04	0.21	0.23 ***
Valence as the Independent Variable and Life Satisfaction as the Dependent Variable				
Valence—Facebook Addiction	0.11	0.07	1.90	0.01
Valence—Stress	−0.04	0.05	−0.69	
Facebook Addiction—Stress	0.21	0.05	3.58 ***	0.04 **
Facebook Addiction—Life Satisfaction	−0.02	0.08	−0.40	
Stress—Life Satisfaction	−0.44	0.09	−8.32 ***	
Valence—Life Satisfaction	0.04	0.09	0.77	0.21 ***
Agency as the Independent Variable and Life Satisfaction as the Dependent Variable				
Agency —Facebook Addiction	−0.39	0.06	−7.32 ***	0.15 ***
Agency —Stress	−0.25	0.05	−4.15 ***	
Facebook Addiction—Stress	0.10	0.05	1.99 *	0.10 ***
Facebook Addiction—Life Satisfaction	0.01	0.08	0.06	
Stress—Life Satisfaction	−0.44	0.10	−7.90 ***	
Agency—Life Satisfaction	0.06	0.09	0.98	0.21 ***
Total effects				
Valence—Psychological Well-Being	0.12	0.05	2.06 *	0.02 *
Agency—Psychological Well-Being	0.21	0.04	3.52 ***	0.05 ***
Valence—Life Satisfaction	0.05	0.09	0.78	0.01
Agency—Life Satisfaction	0.18	0.09	3.18 ***	0.04 **

* *p* < 0.05, ** *p* < 0.01, *** *p* < 0.001.

**Table 3:** Indirect effects and total indirect effects of Facebook addiction and stress in the relationship of social media mindsets (valence and agency) with psychological well-being and life satisfaction (standardized scores).

Pathways		Indirect Effect	SE	LLCI	ULCI
	Valence—Facebook Addiction—Psychological Well-Being	−0.03	0.02	−0.06	0.01
	Valence—Stress—Psychological Well-Being	0.02	0.03	−0.04	0.06
	Valence—Facebook Addiction—Stress—Psychological Well-Being	−0.01	0.01	−0.02	0.001
	Agency—Facebook Addiction—Psychological Well-Being	0.08	0.02	0.03	0.13
	Agency—Stress—Psychological Well-Being	0.10	0.03	0.05	0.16
	Agency—Facebook Addiction—Stress—Psychological Well-Being	0.02	0.01	0.001	0.04
	Valence—Facebook Addiction—Life Satisfaction	−0.01	0.01	−0.02	0.01
	Valence—Stress—Life Satisfaction	0.02	0.03	−0.04	0.08
	Valence—Facebook Addiction—Stress—Life Satisfaction	−0.01	0.01	−0.03	0.002
	Agency—Facebook Addiction—Life Satisfaction	−0.01	0.02	−0.05	0.05
	Agency—Stress—Life Satisfaction	0.11	0.03	0.05	0.18
	Agency—Facebook Addiction—Stress—Life Satisfaction	0.02	0.01	0.001	0.04
Total Indirect Effect					
	Valence—Facebook Addiction—Stress—Psychological Well-Being	−0.02	0.03	−0.09	0.05
	Agency—Facebook Addiction—Stress—Psychological Well-Being	0.19	0.03	0.03	0.13
	Valence—Facebook Addiction—Stress—Life Satisfaction	0.01	0.03	−0.06	0.06
	Agency—Facebook Addiction—Stress—Life Satisfaction	0.13	0.04	0.04	0.21

## Data Availability

The raw data supporting the conclusions of this article will be made available by the authors on request due to its sensitive ethical nature.
